# Effects of the Interlayer Toughening Agent Structure on the Flow Behavior during the z-RTM Process

**DOI:** 10.3390/ma15093265

**Published:** 2022-05-02

**Authors:** Weidong Li, Gang Liu, Jianwen Bao, Shuhua Dong, Xiaolan Hu, Xiaosu Yi, Zhitao Lin

**Affiliations:** 1 Key Laboratory of Advanced Composite, Composite Technology Center, AVIC Composite Corporation Ltd., Beijing 101300, China; liwdhappy@163.com (W.L.); liugang@iccas.ac.cn (G.L.); yi_xiaosu@sina.cn (X.Y.); 2 School of Materials Science and Engineering, Shandong University of Technology, Zibo 255049, China; lin.zhi.tao@163.com; 3 College of Materials, Xiamen University, Xiamen 361005, China; xlhu@xmu.edu.cn

**Keywords:** resin flow, ex situ toughening, permeability, z-RTM process, fabric preforms

## Abstract

In this paper, interlayer toughening composites were prepared by the z-directional injection RTM process (z-RTM), which has the advantage of increasing the interlaminar toughness and shortening the filling time and completely impregnating the fibers. The nonwoven fabrics and dot matrix structure material were used as ex situ interlayer toughening agents. The effect of the interlayer toughening agent structure on the resin flow behavior during the z-RTM process was investigated. The macro-flowing and micro-infiltration behaviors of the resin inside the preforms were deduced. The permeability of the fabric preforms with different toughening agents was investigated. The results show that the introduction of the nonwoven structure toughening agent makes the macro flow slow, and the flow front more uniform. The toughening agent with a dot matrix structure promotes the resin macro flow in the preforms, and shortens the injection time. The z-directional permeability of the preform with a dot matrix structural toughening agent is one order of magnitude lower than that of the non-toughened preform, while being higher than the preform toughened by the nonwoven fabric preforms, which is helpful for the further applicability of the z-RTM process. Furthermore, the mode II interlaminar fracture toughness of composites was evaluated.

## 1. Introduction

Resin transfer molding (RTM), a typical form of liquid composite molding (LCM), has become a mainstream technology because of its low cost, as well as the high performance of the produced composite materials [1,2,3,4,5,6]. The resin can fully infiltrate dry fabric preform by exerting external pressure on a closed mold for the RTM process, which requires an extremely low initial resin viscosity during the injection. Owing to the resin brittleness after solidification, however, the composite materials are prone to exhibiting low damage resistance against low-velocity impact, which leads to delamination during use [7,8,9,10,11]. Therefore, in order to obtain high-performance RTM production, research has to resolve the contradiction between the low resin viscosity and the high toughness of the composites under the condition of constant resin composition, as well as stable chemical and rheological properties [12].

Yi et al. [13] firstly proposed the concept of ex situ toughening, namely, the resin matrix being separated from the toughening agents concentrated in the interlaminar, which is the weakest zone of composites. Previous studies showed that the interlaminar toughness of RTM products was greatly improved using ex situ toughening technology [14,15,16]. Furthermore, the z-directional flow RTM (z-RTM) technology with a resin disperser was put forward in view of increasing the fiber volume fraction, as well as weakening the flow ability because of the introduction of toughening agents [17]. This technology adopted the thickness directional (z-directional) injection method, which enabled the resin flow path to shorten and made resin flow quickly across large areas in the z-direction by increasing the injection pressure and embedding the rapid resin disperser inside the mold. The resin pressure field was formed inside the mold with the same area as the preform surface. In the traditional process of RTM in-plane injection, the flow rate of resin between layers is reduced by the introduction of a toughening agent material between layers, which makes the flow of resin between layers more difficult. The injection process takes a longer time. The technical parameters of the resin initial viscosity and the injection pressure are strict. Compared with the RTM process, the z-RTM process shortens the injection time and, to a great extent, eliminates the difficulties from long-path flowing caused by toughening agents or other functional components.

The permeability of the fabric preform is an important parameter for the design and optimization of the RTM process. More attention has been paid to in-plane permeability (Kx, Ky), which is relevant in practical engineering applications [3,18]. In addition, the permeability of the preform in the thickness direction (Kz, z-direction) has also been evaluated. Lee et al. [19] monitored the resin flow in the z-direction of the preform in real-time and projected the change in the flow front shape based on the fluid equation. Salvatori et al. [20] investigated the mesoscopic pore space of the compacted fabrics, imaged with X-ray tomography, and performed analysis to propose permeability predictions based on the channel’s geometry. The results show that the permeability of two-scale fabric can be accurately measured by unsaturated test without considering the microflow and capillary effect when the capillary number exceeds a certain threshold. Other studies [21] showed that the resin infiltration model was mainly determined by the microstructure of the preform when resin was flowing through the preform in the z-direction. The resin tended to rapidly flow through the gap between the cross-section of fiber bundles in the fabric, as well as completing the infiltration into intra-fibers under the capillary force in the z-direction. Godbole et al. [22] proposed a closed-form permeability model for dual-scale unidirectional (UD) fabric that combines both the tow and inter-tow gap permeability while taking into account the effect of the partially saturated zone during dual-scale flow. Prof. Advani of the University of Delaware [23] studied the flowing infiltration behavior of the resin along the z-direction of the preform. The established flowing model was in agreement with the experimentally measured data.

Overall, the research on the z-directional permeability of fabric preforms and the flow behavior of resin in the z-direction has focused mostly on fabric preforms with simple laminated or 3D woven structures without toughening agents loaded between the fabric layers. To date, few studies have been performed on the influence of interlamination functional components on the flow behavior of the resin. The interlayer toughening agents have an important effect on the resin-filling flow rate and the resin flow modes. The flow of the resin along the z direction makes the resin flow behavior of the process more complicated than that of traditional RTM process. It is key to ensure that the resin is fully infiltrated into the reinforced material in order to obtain a high quality of the product. Therefore, in this paper, the influence mechanism of interlayer toughening agents on the flow behavior of resin, as well as the permeability and the mode II interlaminar fracture toughness of the fabric preforms, was investigated.

## 2. Materials and Methods

### 2.1. Materials

**Figure 1:**
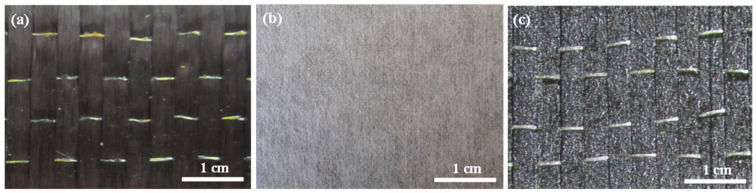
Metallograph of unidirectional carbon fabric and toughening agents. (**a**) Unidirectional carbon fabric; (**b**) nonwoven fabric; (**c**) dot matrix structure.

T700 carbon fiber unidirectional fabrics U7192D were used for fiber reinforcement. Pictures of the structure of the fabric and the toughening agents were taken by metallurgical microscopy (CarlZeiss Axio Scope A1, Oberkochen, Germany), and are shown in Figure 1. The unidirectional carbon fabric is shown in Figure 1a. Two kinds of different toughening agents were used. One of the toughening agents was a form of nonwoven structure (Figure 1b, arial density 20 g/m^2^, thickness 20 μm). The other toughening agent was a form of dot matrix structure, which was preloaded on the surface of the fabrics by the hot melt method (Figure 1c, arial density 20 g/m^2^). Both of the toughening agents were sourced from China AVIC Composite Technology Center. An aqueous solution of malt syrup with a concentration of 55 wt% and a viscosity of 0.05 Pa·s at 25 °C was used as the equivalent resin fluid. For convenience and clarity, the equivalent fluid is referred to as resin throughout the main text.

### 2.2. Fabric Lay-Up and z-RTM Process

**Figure 2:**
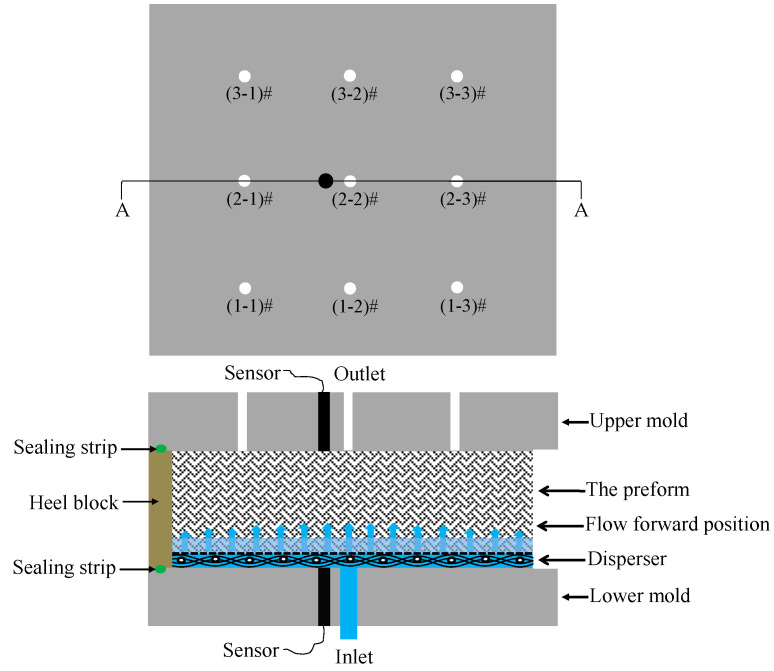
Diagram of pressure monitoring devices for z-RTM process.

The lay-ups of non-toughened preform (NT-Preform), nonwoven-fabric-toughened preform (NWT-Preform) and dot-matrix-structure-toughened preform (DMT-Preform), as well as the injection process parameters, are shown in Table 1. The toughening agent with a nonwoven structure was periodically laid between layer fabrics. The experiments were performed by a new RTM process with z-directional injection. A rapid resin disperser made of stainless steel was embedded at the bottom of mold. The preform was vacuumed for 1 h at 80 °C. The resin was injected into the mold at the center of the lower mold surface using a metering pump at a constant flow of 5 mL/min. There were nine outlets on the upper surface of the mold. The inlet and outlet pressure changes through the gate were monitored using the pressure sensors placed on the upper and lower mold surfaces. The diagram of pressure monitoring devices for the z-RTM process is shown in Figure 2. The efflux time of the resin through each gate was recorded during the experiment. 

### 2.3. Test and Characterization

#### 2.3.1. Fitting of z-Directional Flow Front

The pressure variation laws of the inlet and outlet from the fabric preforms with different toughening agents were monitored by pressure sensors. The resin flow velocity at a different position in the preform was calculated based on the efflux time of the resin through the outlet gate. The flow front at the moment that the resin was flowing out of the middle outlet of the gate was fitted.

#### 2.3.2. Test of z-Directional Permeability

The saturated z-directional permeability was calculated by Darcy’s law. The equation is as follows [1]:

(1) $$\langle u\rangle =-\frac{K}{\mu }\cdot \langle \nabla P\rangle$$

where *u* is the fluid velocity, *µ* is the dynamic viscosity of fluid, *K* is the permeability tensor of porous medium, ∇*P* is the pressure gradient, and < > represents the volume average. According to Equation (1), the equivalent permeability measured by the experiment can be obtained:

(2) $$K_{z}=\frac{Q\cdot \mu \cdot \Delta L}{A\cdot \Delta P}$$

where *K*_z_ is the equivalent saturated z-direction permeability of the preform (m^2^), *Q* is the volumetric flow rate (m^3^/s), Δ*L* is the thickness of the injected preform (m), *A* is the cross-sectional area of the preform (m^2^), and Δ*P* is the pressure difference measured by the pressure sensors (Pa).

The equivalent saturated permeability was tested by the experimental method. The fabric was completely infiltrated by the tested liquid. The pressure at the injection gate was collected. After the outflow liquid reached stability, the volume of the outflow fluid was obtained with a measuring cylinder, and the time was 60 s, so the volume flow per unit time could be calculated. The viscosity of the tested liquid was 0.05 Pa·s. The z-directional permeability could be obtained by the volume flow and pressure gradient from Equation (2).

#### 2.3.3. Test of the Interlaminar Fracture Toughness

The mode II interlaminar fracture toughness (G*_IIC_*) of the laminates was tested by the End Notched Flexure (ENF) tests according to ASTM Standard D7905. The initial delamination length was 45 mm. The loading rate was 1 mm/min. Five specimens were tested for each sample. The mode II interlaminar fracture toughness of laminates, *G_IIC_*, is calculated by Equation (3):

(3) $$G_{IIC}=\frac{3mP_{Max}^{2}a_{0}^{2}}{2B}$$

where *G_IIC_* is the interlaminar fracture toughness of laminates (J/m^2^), *m* is the CC coefficient, *P_max_* is the maximum force (N), *a*_0_ is the crack length used in fracture test (30 mm), and *B* is the specimen width (mm).

## 3. Results

### 3.1. Effect of Toughening Agent Structure on the Resin Injection Pressure

**Figure 3:**
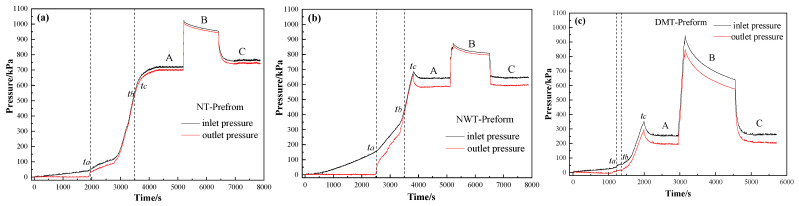
Pressure change laws during z-directional injection. (**a**) NT-Preform; (**b**) NWT-Preform; (**c**) DMT-Preform and stage A means that the inlet open but only outlet of (2-2)# open; stage B means that inlet and all outlets are closed; stage C means that the inlet open but only outlet of (2-2)# open.

**Figure 4:**
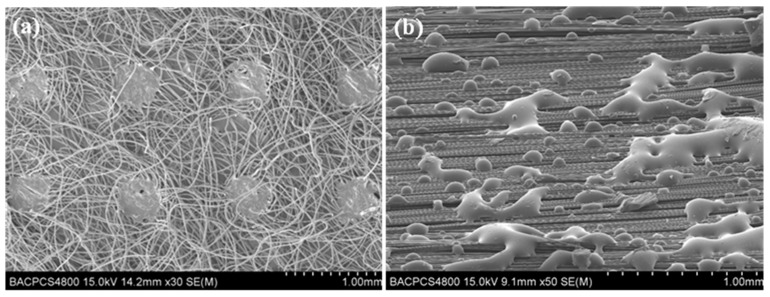
SEM images of toughening agents: (**a**) nonwoven fabric; (**b**) fabric with dot matrix structure.

Figure 3a–c show the pressure change laws of the inlet and outlet at the upper and lower surfaces of the NT-Preform, NWT-Preform and DMT-Preform. It can be seen that the inlet pressure linearly increases with the injection time, while the outlet pressure is zero during 0~*t_a_* time period. At the moment of *t*_a_, the pressure difference between the inlet and outlet gates reaches the maximum of about 40 kPa for the NT-Preform, about 150 kPa for the NWT-Preform and 35 kPa for the DMT-Preform. The pressure difference for the NWT-Preform is higher than that of the NT-Preform and the DMT-Preform. The nonwoven structure layer (see Figure 4a) can be equivalently classified into two parts: low-permeability region (strengthening part) and high-permeability region (outside strengthening part). Upon entering the highly permeable region of the nonwoven structure layer, the resin continues to flow through its pores. The irregularly distributed fiber has the effect of redistributing the micro flow front of the resin. The region of the nonwoven structure with low permeability retards the z-directional resin flow. Therefore, a higher pressure difference is required to drive resin infiltration from low- to high-permeability regions. The pressure difference for the DMT-Preform is lower than that of the NT-Preform and NWT-Preform. The toughening agent enriched part of the DMT-Preform will generate a rapid flowing channel along the inner surface (see Figure 4b). The resin has a lower resistance in the in-plane flow between the layers of the DMT-Preform as compared to the NT-Preforms and NWT-Preforms. The gap layer in the NT-Preform is so small and evenly distributed that it is difficult to generate the in-plane rapid flowing channel. Therefore, the resin in the DMT-Preform requires the lowest driving force for the z-directional unsaturated flow at the early stage.

The outlet is closed while the resin is observed to flow out of the outlet gate during the period from *t*_a_ to *t*_b_, resulting in the continuous increase in pressure at the inlet and outlet gates, as shown in Figure 3a–c. Once the resin flow front arrives at the upper surface of the mold, the corresponding outlet is closed and then the resin front cannot move on in the z-direction. As a result, the resin becomes stagnant, thus causing the fluid pressure to accumulate, which is detected by a pressure sensor at the upper mold surface. The outlet pressure thereby rapidly increases. Then, a larger injection pressure is required for continuous flow through the fully infiltrated region towards the unsaturated region, which also results in a rapid pressure increase at the inlet gate.

All of the outlets are closed at the moment of *t*_b_. The resin is further injected into the mold until time *t*_c_. At time *t*_c_, outlet gate (2-2)# is reopened to keep the flow rate constant inside the preform along the z-direction. At stage A, outlet gate (2-2)# is closed when the pressure of inlet and outlet reaches the pressure balance difference of about 20 kPa for the NT-Preform, 35 kPa for the NWT-Preform, and 58 kPa for the DMT-Preform. Additionally, the inlet is then closed when the injection pressure rapidly increases to 1000 kPa, which causes the resin to further infiltrate the fiber bundles in high-pressure environments.

At stage B, as shown in Figure 3a,b, the pressure difference between the inlet and outlet gates is almost constant, indicating the full infiltration of the resin into the composites during the early unsaturated flowing state. However, the pressure difference between the inlet and outlet gates at stage B for the DMT-Preform reduces from 94 kPa to 65 kPa, suggesting that the resin plays a role in micro-infiltration into the intra-fiber during the constant pressure process. The fiber volume fraction of the DMT-Preform is high at the loaded position, which minimizes the resin’s ease in going through micro-infiltration during the macro flowing stage. Therefore, high pressure and long-term infiltration are required to achieve full micro-infiltration into the intra-fiber around the loaded position. The pressure difference between the inlet and outlet gates at equilibrium remains around 20 kPa for the NT-Preform, 35 kPa for the NWT-Preform and 58 kPa for the DMT-Preform when both inlet and outlet gate (2-2)# are reopened during stage C. This further confirms that the fabric preforms are completely infiltrated. This shows the good condition of the infiltration effect of resin on the fabric preform at the early unsaturated flowing state.

### 3.2. Flow Behavior of Resin in the Preforms

**Figure 5:**
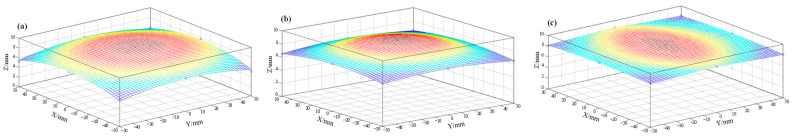
Fitted flow front of resin in the preforms. (**a**) NT-Preform [17]; (**b**) NWT-Preform; (**c**) DMT-Preform.

The recorded resin efflux time through each outlet, that is, the duration from the start of resin injection to the flowing out of each gate, is shown in Table 2. It can be seen that the resin comes out of the outlet gate (2-2)# at first, then the resin is observed to flow out of the other gates in turn. The resin in DMT-Preform flows at the highest speed, and the resin efflux time in the NT-Preform is shorter than that in the NWT-Preform. The geometrical distribution of the flow front surface was fitted according to the observed resin efflux time at the nine gates based on the MATLAB software. The fitting figures of the resin flow front are shown in Figure 5. The resin flow front surface equation of the NT-Preform is as follows, according to Figure 5a:

(4) $$z(x,y)=-1908.6+\frac{1913.7}{1+{\left(\frac{x-1.6}{1856.1}\right)}^{2}}-\frac{325.1}{1+{\left(\frac{y-3.1}{46.8}\right)}^{2}}+\frac{329.1}{\left[1+{\left(\frac{x-1.6}{1856.1}\right)}^{2}\right]\times \left[1+{\left(\frac{y-3.1}{46.8}\right)}^{2}\right]}$$

The flow front surface equation of the NWT-Preform is as follows, according to Figure 5b:

(5) $$z(x,y)=74.1-\frac{86.6}{1+{\left(\frac{x+0.2}{126.3}\right)}^{2}}-\frac{78.3}{1+{\left(\frac{y-0.4}{159.8}\right)}^{2}}+\frac{99.9}{\left[1+{\left(\frac{x+0.2}{126.3}\right)}^{2}\right]\times \left[1+{\left(\frac{y-0.4}{159.8}\right)}^{2}\right]}$$

The flow front surface equation of the DMT-Preform is as follows, according to Figure 5c:

(6) $$z(x,y)=5.2-\frac{259.8}{1+{\left(\frac{x+12.5}{0.8}\right)}^{2}}+\frac{3.2}{1+{\left(\frac{y-1.6}{161.4}\right)}^{2}}+\frac{421.0}{\left[1+{\left(\frac{x+12.5}{0.8}\right)}^{2}\right]\times \left[1+{\left(\frac{y-1.6}{161.4}\right)}^{2}\right]}$$

The resin is injected at the inlet of the mold center and is distributed by the rapid resin disperser. The resin firstly infiltrates the center of the preform, and then gradually spreads to infiltrates the preform. The rigid disperser results in in-plane pressure loss. The pressure at the center position is higher than that in the surrounding area, thereby creating a pressure gradient field that is divergent from center to edge. The resin is observed to firstly flow out of the center of the outlet because of the pressure loss of the disperser and the time difference from the resin contacting the preform. In addition, the pressure loss inside the preform in the thickness direction also occurs, and increases with the increase in the flowing distance of the resin. Therefore, the flow front rapidly spreads from the center of the preform.

It can be seen from Figure 5a,b that the flow front of the NWT-Preform is smoother than that of the NT-Preform. This phenomenon could be caused by two possible reasons. Firstly, the resin has a certain pressure gradient distribution in the rigid disperser, so there is a higher fluid pressure at the center than in the surroundings to form a divergent pressure gradient field. Secondly, the nonwoven toughening layer between the layers in the ex situ toughened preform causes the fiber volume fraction to increase in the preform, which retards the z-directional resin flow. However, high porosity in the nonwoven toughening agent between layers makes the resin prone to diffuse into the region with a lower flow resistance. Therefore, part of the resin at the flow front diffuses along the highly porous interlayer, which can alleviate the phenomenon of the fluid flowing faster at the center than that in the surrounding area. Therefore, the resin flow front in the NWT-Preform was smoother than that in the NT-Preform.

**Figure 6:**
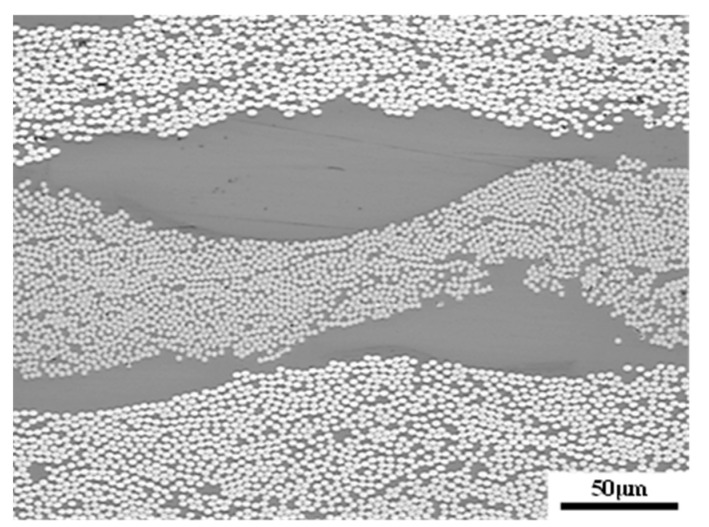
Optical micrograph of distribution and morphology of toughening agent of dot-matrix-toughened structure.

The phenomenon of the flow front diverging from the center to the edge is greatly alleviated in the DMT-Preform (Figure 5c). Although the pressure gradient distribution of the resin occurs in a rigid disperser, the ex situ toughening agent loaded between the layers in the DMT-Preform generates a bulge structure (Figure 6), which leads to a rapid flowing channel between the preform’s layers. As the flow front arrives at the fabric layer, the resin quickly spreads along the in-plane direction. Compared with the NT-Preform and the NWT-Preform, the flow front of the resin in the DMT-Preform is smoother, indicating that the dot matrix toughening agent is helpful for the resin macro flow in the preform, which will reduce injection time and improve injection efficiency.

**Figure 7:**
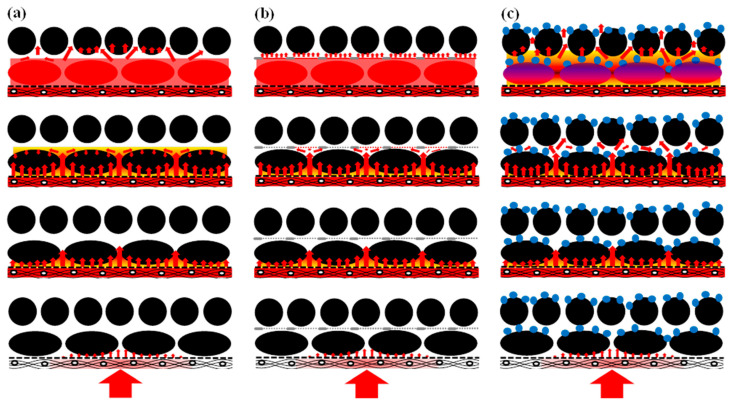
Deduction of z-directional flow and infiltration model. (**a**) NT-Preform; (**b**) NWT-Preform; (**c**) DMT-Preform.

Figure 7 shows the deduction of the z-directional flow and infiltration model. During the z-directional resin-filling process, the resin disperser is firstly filled with the resin under the driving force of external pressure, and then the resin flows along the z-direction to infiltrate the entire preform. The resin firstly flows through the large gap between fiber bundles, which is the region with lower resistance, and then infiltrates into the micro intra-fibers. After the infiltration of the first fabric layer, the second fabric layer is infiltrated with the same method. It can be assumed that the infiltration into more fabric layers can be derived similarly, that is, the in-plane and through-plane infiltration can be derived in a periodical and alternative way, as shown in Figure 7a. It can be seen from Figure 7b that the strengthening point in the nonwoven-fabric-toughened layer delays the resin rapidly flowing along the z-direction when the flow front arrives at the nonwoven-fabric-toughened layer, which facilitates the resin infiltration into the fabrics. In the meantime, the resin flows to the high-permeability region, except for the strengthening part, which further refines the flow front. The resin flows through the high-permeability region in the toughened layer under the driving force of external pressure and starts the z-directional infiltration of the next fabric layer.

It can be seen from Figure 7c that the resin spreads along the rapid flowing channel in the in-plane direction and continues its z-direction filling flow from one layer to the next as the flow front finishes rapidly flowing through the first fabric layer.

### 3.3. Effect of the Structure of Toughening Agents on the z-Directional Permeability of Preforms

**Figure 8:**
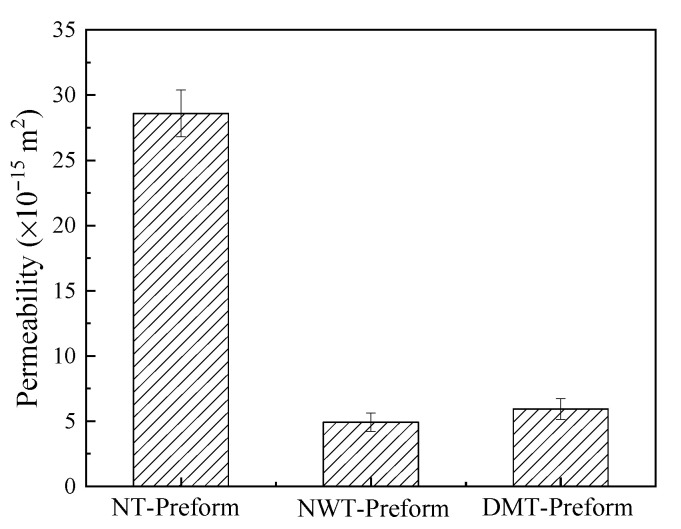
Effects of the toughening agent structures on the z-directional permeability of the preforms.

The effects of the toughening agent structures on the z-directional permeability of the preforms are shown in Figure 8. The z-directional permeability of the NT-Preform is 2.86 × 10^−14^ m^2^, while that of the NWT-Preform is 4.93 × 10^−15^ m^2^, which is 83% lower. The DMT-Preform is 5.94 × 10^−15^ m^2^, which is 79% lower than the NT-Preform. It can be seen that the introduction of the toughening agents reduces the z-directional permeability by one order of magnitude. The z-directional permeability of the NWT-Preform is the lowest among the three preforms. The nominal thickness of the ex situ toughened preform remains unchanged, and the compressibility of nonwoven fabric agents is negligible. Therefore, the introduction of the nonwoven fabrics increases the volume fraction of the single-layer fabric, as shown in the following equation:

(7) $$V_{f}=\frac{N\rho_{A}}{\rho_{f}(H-nh_{film})}$$

where *V*_f_ is the fiber volume fraction, *N* is the number of layers in the fabric, *ρ*_A_ is the areal density of the fabric, *ρ*_f_ is the density of the fabric, *H* is the cavity height, *n* is the number of toughening agent layers, and *h_film_* is the thickness of the toughening agent. Based on the volume fraction calculation for ex situ toughened preform in the nonwoven structures, the volume fraction of the single-layer fiber is about 63.5%. The fiber volume fraction increases; thus, the flow resistance in the z-direction through the preform also increases, and the permeability reduces. The toughening agents of the NWT-Preform result in a uniform resin macro flow front, thus facilitating the successful implementation of the z-RTM process and reducing the probability of forming inner defects. The DMT-Preform has an advantage over the other preform owing to its rapid lead flow diversion, which alleviates the conflict between the resin viscosity and the application of the z-RTM process.

### 3.4. Evaluation of Toughening Effect

**Figure 9:**
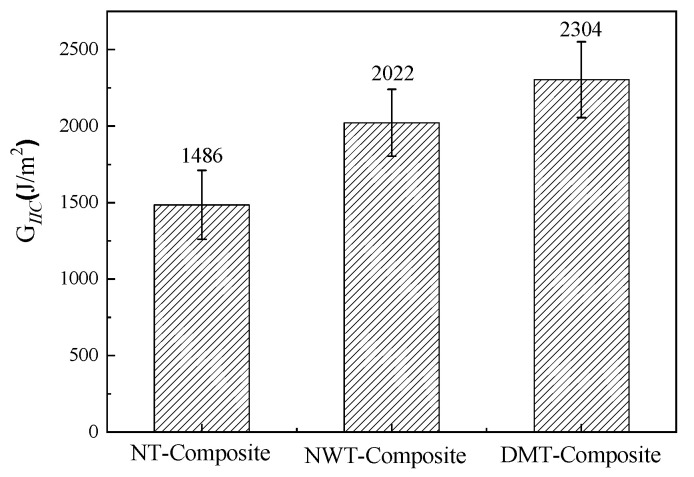
Mode II interlaminar fracture toughness of the three composite laminates.

Figure 9 shows the test results of the mode II interlaminar fracture toughness of the three composite laminates. The mode II interlaminar fracture toughness of the nonwoven-fabric-toughened composite (NWT-Composite) is about 36% higher than that of the non-toughened composite (NT-Composite), and the mode II interlaminar fracture toughness of the dot-matrix-toughened composite (DMT-Composite) increased by 55% compared with the NT-Composite. Both toughening structures have obvious effects when it comes to improving the mode II interlaminar fracture toughness of composites.

**Figure 10:**
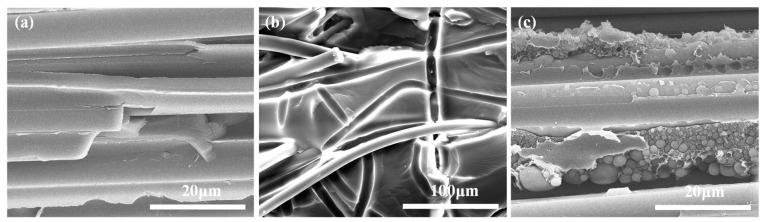
Comparison of the interlaminar failure morphologies among three G*_IIC_* samples after etching. (**a**) NT-Composite; (**b**) NWT-Composite; (**c**) DMT-Composite.

Figure 10 shows the comparison of the interlaminar failure morphologies among three *G_IIC_* samples after etching. It can be seen that the fracture of the interlaminar resin of NT-Composite samples presents the typical brittle failure characteristics of the *G_IIC_* samples (Figure 10a). It can be seen from Figure 10b that the structural form of the nonwoven fabric in the curing process of the NWT-Composite is retained. Under the action of mode II shear stress, the plastic deformation, fracture and pull-out of thermoplastic fiber absorbs a large amount of cracking energy, which plays a role in hindering crack propagation. Therefore, the mode II interlaminar fracture toughness is greatly improved. It can be seen from Figure 10c that the DMT-Composite toughening agent and the resin undergo reaction-induced phase separation, forming a thermoplastic-thermosetting bicontinuous phase structure. The interlaminar phase structure effectively improves the mode II interlaminar fracture toughness of the composites.

## 4. Conclusions

The effect of the interlayer toughening agent structure on the resin flow behavior during the z-RTM process was investigated. The results show that the introduction of nonwoven fabrics as toughening agents reduces the macro flow rate and creates a uniform resin flow front and a smooth flow front surface. The toughening agent with a dot matrix distribution facilitates the macro flow of the resin in the preform, thus reducing the injection time and improving injection efficiency as compared with the NT-Preform. The z-directional permeability of the NWT-Preform and DMT-Preform is 4.93 × 10^−15^ m^2^ and 5.94 × 10^−15^ m^2^, respectively, which is about one order of magnitude lower than that of the NT-Preform. The use of toughening agents with a dot matrix structure has better applicability for the z-RTM process. The mode II interlaminar fracture toughness of nonwoven-fabric-toughened composite and dot-matrix-toughened composite increased by 36% and 55% over non-toughened composite, respectively.

## Figures and Tables

**Table 1:** Lay-up sequence and manufacturing process of unidirectional carbon fabric.

Samples	Stacking Sequence	Thickness/mm	Fiber Volume Fraction/%	Preforming Parameters
NT-Preform	[45°/0°/−45°/90°]_6S_	9	57.5	Vacuumed for 1 h at 80 °C
NWT-Preform	[45°/0°/−45°/90°]_6S_	9	57.5	Vacuumed for 1 h at 80 °C
DMT-Preform	[45°/0°/−45°/90°]_6S_	9	57.5	Vacuumed for 1 h at 80 °C

Note: NT-Preform means non-toughened preform; NWT-Preform means nonwoven fabric-toughened preform; DMT-Preform means dot-matrix-structure-toughened preform.

**Table 2:** Efflux time of resin through each outlet.

Time (s)	(1-1)#	(2-1)#	(3-1)#	(1-2)#	(2-2)#	(3-2)#	(1-3)#	(2-3)#	(3-3)#
NT-Preform	3384	2472	2976	2448	1950	2292	3108	2574	3492
NWT-Preform	3486	3168	3450	3114	2502	3138	3468	3192	3474
DMT-Preform	1363	1278	1345	1302	1224	1315	1345	1290	1368

## Data Availability

Not applicable.
